# Editorial: Gene therapy for the central and peripheral nervous system, volume II

**DOI:** 10.3389/fnmol.2023.1258458

**Published:** 2023-08-01

**Authors:** Andrew P. Tosolini, George M. Smith

**Affiliations:** ^1^Department of Neuromuscular Diseases, Queen Square Institute of Neurology, University College London, London, United Kingdom; ^2^UCL Queen Square Motor Neuron Disease Centre, University College London, London, United Kingdom; ^3^Australian Institute for Bioengineering and Nanotechnology, The University of Queensland, St Lucia, QLD, Australia; ^4^School of Biomedical Sciences, Faculty of Medicine, The University of Queensland, St Lucia, QLD, Australia; ^5^Shriners Hospitals Pediatric Research Center, Lewis Katz School of Medicine, Temple University, Philadelphia, PA, United States; ^6^Department of Neural Sciences, Lewis Katz School of Medicine, Temple University, Philadelphia, PA, United States

**Keywords:** gene therapy, AAV, miRNA, CNS, PNS, transduction, lncRNA, circRNAs

After the great success of the first volume, we are proud to present *Volume II* of this specialized Research Topic on *Gene therapy for the central and peripheral nervous system*. *Volume I* discussed gene therapies for the treatment of diverse neurodegenerative disorders including spinocerebellar ataxia, spinal muscular atrophy (SMA), amyotrophic lateral sclerosis (ALS), as well as disorders of the central nervous system (CNS) and peripheral nervous system (PNS), including, stroke, peripheral nerve repair and neuropathic pain (Tosolini and Smith, 2018). *Volume II* has expanded upon these ideas and concepts, and has also included submissions focused on the basic pathomechanism(s) of various CNS/PNS disorders, with the intention to include conversations of target identification and validation for the development of future gene therapies.

The aim of most gene therapy pipelines is to transition successful and meaningful outcomes from the pre-clinical experimental setting, regardless of the model system, into viable treatment options for patients (Zhao et al., 2022). As a scientific community, we should be immensely encouraged with the recent triumphs of the clinically available gene therapies, such as Luxturna for RPE65-associated retinal dystrophy (Smalley, 2017), as well as Zolgenmsa (Mendell et al., 2017) and the antisense oligonucleotide (ASO) therapy Nusinersen (Mercuri et al., 2018) to treat *SMN1*-linked SMA. However, these monumental accomplishments have come from treating monogenic disorders, and harnessing those successes to treat more complicated afflictions of the CNS/PNS (e.g., ALS, spinal cord injury, etc.) appear as the next major challenge for the field (Tosolini and Sleigh, 2017).

Nevertheless, there is great excitement in expanding the list of clinically available gene therapies, with the continuous development and optimisation of technologies that result in better outcomes (Challis et al., 2022). Indeed, novel viral serotypes and capsids are being improved (Chan et al., 2017; Andari et al., 2022; Goertsen et al., 2022), and in combination with tissue/cell specific promoters (Wang et al., 2008; Jonquieres et al., 2013; Nieuwenhuis et al., 2021), enable a more exquisite targeting of select populations of subsets of neurons/cells, which reduce the potential of detrimental off-target effects. At the same time, novel ASOs and ASO-like molecules are also being developed and combat disease by altering gene expression through interactions with specific regions of the targeted gene with the ultimate goal of supressing pathological protein load (Fletcher et al., 2016).

To complement the advances in gene therapy bioengineering, developments in assistive technology, such as focused ultrasound methods (Blesa et al., 2023), are being implemented to improve blood-brain-barrier permeability and augment access of gene therapies to targeted CNS/PNS regions. Furthermore, novel delivery methods, such as subpial injections, have also been implemented to enhance therapeutic efficacy of gene therapies, and are being applied to small and large mammals, such as mice and non-human primates (Bravo-Hernandez et al., 2020). Alternatively, as viral-vectors can be internalized in axon terminals and undergo retrograde axonal transport in motor and sensory neurons, administering gene therapies to skeletal muscle is a minimally invasive approach to transduce the CNS (Tosolini and Sleigh, 2020). On the other hand, combining muscle-specific viral-serotypes with promoters will restrict transduction to skeletal muscle, but can therapeutically influence the innervating neuronal populations (Sleigh et al., 2023).

We launched the second installment in 2021 and we are proud to showcase the ten publications in this Research Topic that are comprised of six original research articles (Table 1) and four review articles (Table 2).

**Table 1:** A summary of the key findings of each *Original* article published in this Research Topic.

**Article**	**Highlights**	**Type**
Fröhlich et al.	1. Designed and tested a dual-function AAV vector that increased *ASPA* expression whilst lowering *Nat8l* expression in *in vitro* and *in vivo* experimental settings. 2. Post-symptomatic treatment robustly improved disease phenotypes of aged Canavan diseased mice. 3. Concluded that using such dual-function vectors can create a bigger therapeutic window for Canavan disease, and has implications for other leukodystrophies.	Original research
Liu and Rask-Andersen	1. Performed an RNAscope^®^ analyses to spatially localize targeted mRNAs in specific human cochlea regions. 2. Visualized single gene transcripts in the human cochlea using super-resolution structured illumination microscopy (SR-SIM). 3. Identified that most Na/K-ATPase gene transcripts were localized in specialized areas of the cochlear wall epithelium, fibrocyte networks, and spiral ganglions to confirm their essential role in human cochlear function.	Original research
Surdyka et al.	1. Compared the transduction efficiencies of AAV-PHP.eB and AAVrh10 in the cerebellum. 2. Revealed that CMV and PGK promoters induced comparable transduction levels in Purkinje and granule cells. 3. Demonstrated that the location of injections (i.e., deep cerebellar nuclei vs lobular injections) induced different patterns of transduction of cerebellar neurons.	Original research
Arizaca Maquera et al.	1. Analyzed the transcriptome of the entorhinal and temporal cortices during progression of Alzheimer's disease (AD) using human postmortem samples from patients with Braak stages I–VI. 2. Indicated that during disease progression circRNAs could act on the protein level after their translation is activated through A > I RNA editing. 3. Proposed a novel role of circular RNAs in AD.	Original research
Baker et al.	1. Examined the effects of paclitaxel (PTX) on the voltage-gated sodium channel Na_V_1.8 in dorsal root ganglion (DRG) neurons cultured in microfluidic chambers. 2. Using Optical Pulse-chase Axonal Long-distance microscopy, PTX treatment elevated Na_V_1.8 levels in DRG axon tips, and enhanced flux of Na_V_1.8 containing vesicles. 3. Highlighted the importance of subcellular localization of ion channels in DRG neurons in the context of chemotherapy induced peripheral neuropathy.	Original research
Li et al.	1. Established an experimental rat model of Parkinson's Disease (PD) using Rotenone-induced lesions to the striatum. 2. Applied next generation sequencing (NGS) to characterize the expression profile of striatal miRNAs in control and PD-induced rats. 3. Identified therapeutic targets by evaluating the miRNA profiles of extracellular vesicles from midbrain regions of control and PD-rats.	Original research

**Table 2:** A summary of the key findings of each *Review* article published in this Research Topic.

**Article**	**Highlights**	**Type**
Ding et al.	1. Described the mechanisms of the cGAS–STING pathway, and how it is altered in various disorders of the CNS. 2. Identified the different viral proteins that suppress the cGAS-STING signaling pathway. 3. Listed the therapeutic approaches that have been used to target the cGAS-STING signaling pathway in different CNS disorders.	Review
Ni et al.	1. Detailed the pathological mechanisms associated with aging-related neurodegenerative diseases. 2. Discussed the role of long non-coding RNAs (lncRNAs) in aging. 3. Described the link between lncRNAs with neurodegenerative diseases, such as Alzheimer's Disease, Parkinson's Disease, Huntingtons Disease and Amyotrophic Lateral Sclerosis.	Review
Zhou et al.	1. Summarized the different routes of gene therapy administration of rAAVs to the rodent CNS. 2. Identified the anterograde and retrograde axonal transport of various rAAVs in different regions of the brain. 3. Concluded that the administration route of individual rAAVs, along with the choice of promoter, must be carefully considered when targeting specific brain regions, and how that the avenue in which rAAVs can be delivered might be compromised in CNS disorders.	Review
Fischer et al.	1. Compared the differences between the *C. elegans, Drosophila melanogaster*, zebrafish and mouse models that have been used in epilepsy research. 2. Described the pharmaceutical, genetic and optogenetic tools to manipulate the *Drosophila melanogaster* for epilepsy research, and listed the behavioral, electrophysiological, and imaging methods available to assess functional alterations caused by mutations in genes causing epilepsy. 3. Discussed the newly established methods to enable a high-throughput platform for the development of novel precision medicine therapeutics.	Review

We would like to thank all the authors who have contributed to the continued important discussion on gene therapy to treat the CNS/PNS.

## Author contributions

All authors listed have made a substantial, direct, and intellectual contribution to the work and approved it for publication.
